# Why Do People Divorce Late in Life? Swedish Gray Divorce Narratives

**DOI:** 10.1093/geroni/igab046.1527

**Published:** 2021-12-17

**Authors:** Peter Öberg, Torbjorn Bildtgard

**Affiliations:** 1 University of Gävle, Gävle, Gavleborgs Lan, Sweden; 2 Stockholms University, Stockholm, Stockholms Lan, Sweden

## Abstract

Divorce rates for people 60+ has increased in many parts of the Western world in what has been described as a “grey divorce revolution”. In Sweden these divorce rates have more than doubled since the millennium. But why do people choose to divorce late in life and what is the impact of life phase typical transitions? Qualitative interviews with 37 Swedish men and women (aged 62-81) divorced after the age of 60 were collected, covering themes regarding the divorce process: motives for and experiences of divorce, and life as grey divorcee. The results by thematic analysis show that motives for divorcing earlier in the life-course, such as abuse, unfaithfulness and addiction are prevalent also among older people. However, they tend to be framed differently in later life and be integrated into divorce narratives informed by age. We identified four life phase typical narratives for divorce: 1) Lack of a common project for the third age. 2) Partners personality change due to age related disease. 3) Increased freedom after empty-nest allowing emancipation from a dominant partner. 4) A final romantic adventure as a form of rejuvenation. All these life-phase typical narratives are related to the third age as a time of self-fulfillment, where the partner can either be part of or an obstacle to that project. The results will be used to discuss current older cohorts’ views of family norms and later life from the perspective that current older cohorts participated in the divorce revolution in the 1970s as young adults.

